# Complete Genome Sequence of Pseudomonas sp. Strain 273, a Haloalkane-Degrading Bacterium

**DOI:** 10.1128/mra.00176-23

**Published:** 2023-06-08

**Authors:** Yongchao Xie, Gao Chen, Diana Ramirez, Jun Yan, Frank E. Löffler

**Affiliations:** a Department of Civil and Environmental Engineering, University of Tennessee, Knoxville, Tennessee, USA; b Center for Environmental Biotechnology, University of Tennessee, Knoxville, Tennessee, USA; c Department of Microbiology, University of Tennessee, Knoxville, Tennessee, USA; d Key Laboratory of Pollution Ecology and Environmental Engineering, Institute of Applied Ecology, Chinese Academy of Sciences, Shenyang, Liaoning, China; e Department of Biosystems Engineering and Soil Science, University of Tennessee, Knoxville, Tennessee, USA; f Biosciences Division, Oak Ridge National Laboratory, Oak Ridge, Tennessee, USA; Montana State University

## Abstract

Pseudomonas sp. strain 273 utilizes terminally mono- and bis-halogenated alkanes (C_7_ to C_16_) as carbon and energy sources under oxic conditions. During metabolism of fluorinated alkanes, strain 273 releases inorganic fluoride and synthesizes fluorinated phospholipids. The complete genome sequence consists of a circular 7.48-Mb chromosome with a G+C content of 67.5%, containing 6,890 genes.

## ANNOUNCEMENT

Synthetic halogenated alkanes have broad utility, but many are toxic (1, 2), with per- and polyfluoroalkyl substances (PFAS) and their breakdown products of particular concern (3). Pseudomonas sp. strain 273, isolated from garden soil collected in Stuttgart, Germany, utilizes terminally mono- and bis-chlorinated and fluorinated medium- and long-chain alkanes (C_7_ to C_16_) as the sole carbon and energy sources under oxic conditions (4–6). Nonstoichiometric fluoride release was observed during growth with 1,10-difluorodecane because the bacterium channels fluorinated metabolites into phospholipid biosynthesis (7). Taxonomic profiling grouped strain 273 in the genus Pseudomonas (4), and whole-genome average nucleotide identity (ANI) analysis revealed high similarity with Pseudomonas delhiensis (ANI, 97.8%) and Pseudomonas citronellolis (ANI, 94.61%).

Strain 273 was retrieved from a glycerol stock and cultivated at 30°C and 120 rpm in 160-mL glass bottles containing 50 mL of defined basal salt medium amended with 1-fluorodecane (4–6). Genomic DNA of strain 273 was extracted using a cetrimonium bromide-based method (8) and sequenced (PacBio RS II platform; Pacific Biosciences, CA). A long-insert DNA library, with an average fragment size of 20 kb, was prepared by shearing the DNA using a g-TUBE device (Covaris, MA). Hairpin adapters were ligated to fragmented DNA using the SMRTbell template preparation kit (Pacific Biosciences). Library size selection was performed using the BluePippin system (Sage Science, MA), followed by sequencing on a single-molecule real-time cell using PacBio P6-C4 chemistry with one 240-min movie. The 84,871 PacBio reads represent a total length of 887,464,747 bp, with an *N*_50_ read length of 14,826 bp. Quality control of the raw data and the genome assembly were performed using the HGAP (SMRT Analysis v.2.3.0), Canu v.1.2 (9), and Celera v.8.2 assemblers with default parameters. The assemblies were checked using NUCmer v.3.0 (10) and visualized with Circleator (11). The single-contig consensus genome had 107.6-fold coverage and was polished using Quiver (SMRT Analysis v.2.3.0). The JGI IMG v.4.16.6 annotation pipeline (12) and NCBI Prokaryotic Genome Annotation Pipeline (PGAP) (13) were used for gene calling and functional annotation.

The complete genome of strain 273 comprises a circular chromosome of 7,477,410 bp with a G+C content of 67.5%. The genome harbors 6,890 genes, including 6,758 putative protein-coding genes, 66 tRNA genes, and 5 gene clusters, each containing 5S, 16S, and 23S rRNA genes. Strain 273 is a complete denitrifier, and the genome encodes the canonical denitrification pathway, including six *narGHI* genes, one *nirK* gene, three *norBCZ* genes, and one clade I *nosZ* gene. No *nrfA* genes were present. Three alkane 1-monooxygenase (*alkB*) genes, five cytochrome P450 (CYP) genes, and one haloalkane dehalogenase (*hkd*) gene were identified with potential roles in (halo)alkane catabolism. Genes for rhamnolipid surfactant production were absent, but genes for chemotactic response and motility were present, presumably relevant for accessing non-aqueous phase substrates. The genome of Pseudomonas sp. strain 273 represents a novel resource for studying bacterial (halo)alkane metabolism.

### Data availability.

The raw reads and the complete genome sequence of Pseudomonas sp. strain 273 have been deposited at GenBank (accession number CP116775) and in the Sequence Read Archive (accession number SRR24356093). The JGI IMG annotated genome is available under the name Pseudomonas sp. strain 273 with the IMG genome ID 2814122901.
